# Association of psychosis and oral health: case-control study

**DOI:** 10.1007/s00784-025-06463-6

**Published:** 2025-08-06

**Authors:** Uta Christine Wölfle, Franziska Beck, Nils Werner, Vinay Pitchika, Katrin Heck, Matthias Folwaczny, Falk Schwendicke, Emanuel Boudriot, Sophie-Kathrin Greiner, Alkomiet Hasan, Peter Falkai, Lisa Löhrs, Caspar Victor Bumm

**Affiliations:** 1 Department of Conservative Dentistry and Periodontology, University Hospital, LMU Munich, Goethestraße 70, Munich, 80336 Germany; 2 Department of Psychiatry and Psychotherapy, University Hospital, LMU Munich, Munich, Germany; 3 Max Planck Institute of Psychiatry, Munich, Germany; 4 Department of Psychiatry, Psychotherapy and Psychosomatics, Medical Faculty, University of Augsburg, Augsburg, Germany; 5 German Center for Mental Health (DZPG), partner site Munich/Augsburg, Augsburg, Germany

**Keywords:** Psychotic disorders, Schizophrenia, Psychopharmacology, Oral diagnosis, Dental caries, Holistic health

## Abstract

**Objectives:**

Roughly one in eight individuals presents with psychiatric disorders which were proposed to significantly affect oral health. This study compared oral health of 112 patients (mean 28 years), 31 with schizophrenia spectrum disorders (SSD) or 33 with major depression/bipolar disorders (MDD/BD) to 52 healthy controls (HCG).

**Materials and methods:**

Oral health parameters, including caries experience (decayed-missed-filled teeth/surfaces-index DMFT/DMFS), the presence of plaque (plaque-index PI) and periodontal health (bleeding on probing BOP, periodontal probing depths PPD%), were evaluated by examiners blinded to psychiatric diagnoses. Descriptive statistics summarized demographic and clinical data. Group differences and associations with dental outcomes were analyzed using Chi-square or Mann-Whitney-U tests. Logistic regression identified predictors of oral health.

**Results:**

Patients with SSD or MDD/BD resented with significantly oorer oral health than HCG regarding DMFT (SSD = 9; MDD/BD = 10; HCG = 2), DMFS (SSD = 10; MDD/BD = 12; HCG = 1), PI (SSD = 2; MDD/BD = 2; HCG = 1), BOP (SSD = 20%; MDD/BD = 17%; HCG = 3)% and PPD% (SSD = 1%; MDD/BD = 0%; HCG = 0%) and smoked more often. Differences regarding dental anxiety were not significant (*p* = 0.112). Subgroup analysis showed no differences between SSD and MDD/BD.

**Conclusions:**

SSD and MDD/BD and smoking are key contributors to poor dental health shown by significantly worse DMFT, DMFS and PI, BOP. Probably this might additionally be enhanced by concomitant medication, with multiple psychiatric medication being associated with poorer oral health regarding DMFT, PI and BOP.

**Clinical relevance:**

Routine dental care and personalized oral hygiene training to address disease-specific risks are desirable for individuals with SSD or MDD/BD.

**Supplementary Information:**

The online version contains supplementary material available at 10.1007/s00784-025-06463-6.

## Introduction

Affective and non-affective psychoses are severe psychiatric disorders that often manifest already in early adulthood [1–4]. These encompass a range of complex pathological conditions, including affective disorders such as major depressive disorder (MDD) and bipolar disorder (BD), as well as severe psychotic disorders like schizophrenia spectrum disorder (SSD). These conditions have profound and lifelong impact on patients’ psychosocial well-being, often acting as risk factors for further diseases [5].

Oral health, a critical component of general health, strongly correlates with overall health behavior and provides a straightforward approach to assess the patients’ attitude towards self-care practices [6]. As shown before, psychiatric disorders can significantly affect oral health due to the condition itself, associated self-care deficits or side effects of medications [7–9], which in turn negatively impacts on oral-health-related quality of life [10–14] and treatment needs [7, 12, 14–16]. Previous studies reported an increased caries experience in patients with SSD [13, 15, 17–20], while the overall evidence supporting such association remains highly uncertain [15]. Similarly, periodontal treatments needs were reported to be increased in patients with a psychosis history [7, 8, 21] specifically in SSD [14, 22–24]. Generally, cofactors such as age [25], duration of SSD [25, 26] or tobacco use [21, 25] or the intake of antipsychotic medications might additionally contribute to poorer oral health [26–28]. Notably, most previous data are based on middle-aged or older patient cohorts [13, 17–20, 24, 29], sometimes exclusively considering either male or female patients [24, 29] or cases with complex prosthodontic rehabilitation [23].

The present case-control study aimed to examine oral health in patients with SSD and MDD/BD compared with a control group, and to identify the impact of cofactors as smoking, medications and dental anxiety on oral health. Our null hypothesis was that non-affective psychosis (SSD) or affective psychosis (MDD/BD) alone or together with smoking, dental anxiety or medications were not associated with poorer oral health.

## Materials and methods

### Ethical approval statement

The study was conducted in accordance with the ethical guidelines of the Helsinki Declaration and was approved by the Ethics Committee of the Medical Faculty of the Ludwig-Maximilians-University (No. 18–495). All study subjects gave written informed consent prior to their enrollment into the study.

### Study design and setting

Reporting of this study follows the guidelines for *Strengthening and Reporting of observational Studies in Epidemiology*, STROBE. The study was conducted as a monocentric, prospective case-control study, with participants being enrolled between December 2018 and September 2021, mainly from Munich and the surrounding Bavarian areas. A total of 112 cases with a diagnosis of SSD or MDD/BD were recruited from the patient pool of the Department of Psychiatry and Psychotherapy at LMU. Additionally, 56 mental healthy controls (HCG) were recruited from the patients pool at the Department of Conservative Dentistry and Periodontology at LMU and assessed for mental health by the Department of Psychiatry and Psychotherapy (**SI 1**). Recruitment took place in both departments with procedures adapted by the departments specifically regarding the chronological order, with participants either first completing a questionnaire or receiving a dental check-up as primary step. However, all participants completed both steps.

### Study population

The inclusion criteria for the study were as follows: (1) age between 18 and 65 years. We set the upper age limit of 65 years mainly to reduce confounding from comorbidities and polypharmacy and to minimize logistical challenges. (2) diagnosis of psychiatric disorder, i.e., SSD or MDD/BD for the study group. For the healthy control study patients: (1) age between 18 and 65 years with a preference for patients in their early twenties until early thirties as the typical onset time for psychotic disorders [30] for matching the prospective age of the study group. (2) Absence of latent psychiatric disorders, which was asked generally, and which was confirmed later through screenings that included a questionnaire and a preliminary telephone interview, followed by thorough evaluations conducted by the Department of Psychiatry and Psychotherapy.

### Psychological variables

Data collection involved the administration of various questionnaires to evaluate the mental health, i.e. Positive and Negative Syndrome Scale (PANSS) [31], Hamilton Depression Scale (HAMD) [32], as well as a telephone interview conducted by the Department of Psychiatry and Psychotherapy (SKG, LL). These instruments were used to gather data on sociodemographic characteristics, general and mental health status, activity levels, subjective quality of life, and sexual health. For this study, the psychological questionnaire included questions about age, age at diagnosis, sex, actual tobacco use/smoking (yes/no), modified dental anxiety scale (MDAS), psychiatric disorders (SSD, MDD/BD) and related medications specifically considering typical and atypical antipsychotics, antiepileptics, antidepressants, mood stabilizers, and their combinations in German language.

### Dental variables

Clinical dental examination was conducted at the Department of Conservative Dentistry and Periodontology, LMU, under corrected artificial light, using plane mouth mirrors, WHO CPI and UNC15 probes. Using the WHO assessment system [33] two experienced dentists (UW, CB), calibrated in advance for consistency (interrater reliability: κ = 0.83, calibrated on 10 patients not included the study for periodontal probing), being blinded of the psychiatric diagnoses performed the dental examinations. These yielded DMFT/DMFS index and Quigley Hein Plaque Index (PI). A comprehensive periodontal assessment at six sites per tooth yielded the proportion of sites with PPD > 3 mm (PPD%), the percentage of sites with bleeding on probing (BOP).

### Source of Bias

During dental examination, the specific psychiatric diagnosis was not disclosed, although examiners were aware that patients in the experimental group provided a relevant psychiatric disorder, potentially introducing bias into the dental data. The healthy control group was selected based on the absence of a history of psychiatric conditions, which was later additionally confirmed by the Department of Psychiatry and Psychotherapy. Moreover, the significant dropout of patients unwilling to undergo dental exams also might have caused a positively biased selection.

### Sample size

As the dental analysis constitutes a subgroup analysis, no *a *priori sample size was determined for this analysis. Consequently, post -hoc power was calculated using G*Power (version 3.1) based on the R Squared values obtained from the linear regression model of DMFT scores (0.230). This analysis assesses the overall effect of mental disorders on DMFT. In accordance with established conventions for multiple regression models, the analysis incorporated additional parameters, namely the number of predictors, a significance level of 0.05, and the total sample size of the study. The study demonstrated a calculated power of 0.99.

#### Statistical analysis

Normality of data was tested using Kolmogorow-Smirnow-test. Non-normally distributed data are presented as median and interquartile range [q1-q3] and categorical variables as relative frequencies (percentage). For DMFT and DMFS, means with standard deviations were additionally reported despite their non-parametric distributions to allow for better comparison with other studies that commonly use mean values. Chi-squared-test (χ²-test) was used to test for independency of data, differences between groups were compared using Mann–Whitney *U* test or Kruskal-Wallis test with Dunn-Bonferroni *post-hoc* test for non-normally distributed data and the Chi-squared test for categorical variables. Linear regression analysis was performed to delineate associations between psychiatric conditions and oral health indices adjusting for the co-variates sex and smoking. The corresponding data are presented as β-coefficients with 95% confidence interval (CI). *p*-values ≦ 0.05 were considered significant. All analyses were performed using SPSS (Version 29.0; IBM, Corp., New York, USA), Excel (version 16.90.2; Microsoft, Redmond, USA) and Affinity Designer (Serif, Nottingham, UK).

## Results

### Study population/participants

168 patients were identified after assessing for eligibility. The final study sample comprised 112 subjects with a diagnosis of SSD or MDD/BD and 56 mentally healthy controls. 6 participants were excluded due to incomplete data, 42 declined a dental checkup, 4 of the HCG were later diagnosed to have psychiatric disorders (**SI 1**).

### Descriptive data

The study sample comprised 31 patients with SSD (median: age = 33 (24;42) y; age of diagnosis = 21 (18; 34) y; duration of illness = 11 (3;18) y), 33 patients with MDD/BD (median: age = 34 (26; 49) y; age of diagnosis 18 (12; 27) y; duration of illness = 10 (7;17) y) and 52 mental healthy control individuals (median: age = 26 [23; 28] y). The male to female ratio was comparable among study groups SSD (38.7%), MDD/BD (42.4%) and HCG (53.8%) (*p* = 0.349). Smoking was significantly different with a higher relative amount of smokers in the SSD group (45.2%) and the MDD/BD group (45.5%) as compared to the healthy controls (9.6%). The modified dental anxiety scale identified 4 participants with high dental anxiety (MDAS ≥ 19), 2 among SSD patients and 2 among the MDD/BD group (Table 1). The majority of mentally diseased patients (N = 36) were under complex medication (more than one psychotropic substance), while 16 used a single medication only, 12 patients provided incomplete medication data.

**Table 1:** Characteristics of the study population and oral health parameters

	Variable	All	SSD	MDD/BD	HCG	*p*-value
	Individuals [N]	116	31	33	52	
Characteristics	Age [years]^1^	28 (29;32)	33 (24;42)	34 (26;49)	26 (23;28)	**0.002**
	Age at diagnosis [years]^1^	20 (18;29)	21 (18;34)	18 (2;27)	-	-
	Duration of illness [years]^1^	11 (4;18)	11 (3;18)	10 (7;17)	-	-
	Female [N(%)]	54 (47)	12 (39)	14 (42)	28 (54)	0.349
	Currently smoking [N(%)]	34 (29)	14 (45)	15 (46)	5 (10)	**< 0.001**
	MDAS [N (%)]	4 (3.4)	2 (7)	2 (6)	0 (0)	0.112
Oral health parameters	Median DMFT^1^	7 (1;13)	9 (5;17)	10 (4;17)	2 (0;8)	**< 0.001**
	Median DT^1^	0 (0;3)	1 (0;5)	1 (0;3)	0 (0;0)	**0.002**
	Median MT^1^	0 (0;1)	0 (0;2)	0 (0;2)	0 (0;0)	**< 0.001**
	Median FT^1^	4 (1;9)	6 (2;9)	6 (1;10)	2 (0;7)	**0.018**
	Median DMFS^1^	5 (0;23)	10 (4;38)	12 (2;36)	1 (0;7)	**< 0.001**
	Mean DMFT^2^	8 ± 8	11 ± 8	11 ± 8	5 ± 6	
	Mean DMFS^2^	15 ± 23	21 ± 22	23 ± 30	7 ± 14	
	PI [grade]^1^	1 (1;2)	2 (2;3)	2 (1;2)	1 (1;1)	**< 0.001**
	BOP [%]^1^	9 (3;24)	20 (9;28)	17 (10;39)	3 (0;8)	**< 0.001**
	PPD% [%]^1^	0 (0;1)	1 (0;4)	0 (0;1)	0 (0;0)	**< 0.001**

SSD = schizophrenia spectrum disorder, CDD/BD = major depression disorder/bipolar disorder, HCG = healthy control group, MDAS = modified dental anxiety scale. DMFT/DMFS = decayed-missed-filled index for teeth/surfaces, PI = plaque index, BOP = bleeding on probing, PPD% = proportion of periodontal pockets in %

^1^ Data shown as medians with (25;75 percentile) following non normality distribution tested by Kolmogorow-Smirnow-test

^2^ Data shown as means with standard deviation to enhance comparability with findings from other studies

Pearson-χ²-test for the variables female and currently smoking presented as N (%). Fisher-Freeman-Halton test for MDAS: N = 86 (reason: N = 30 form not completed)

**Fig. 1:**
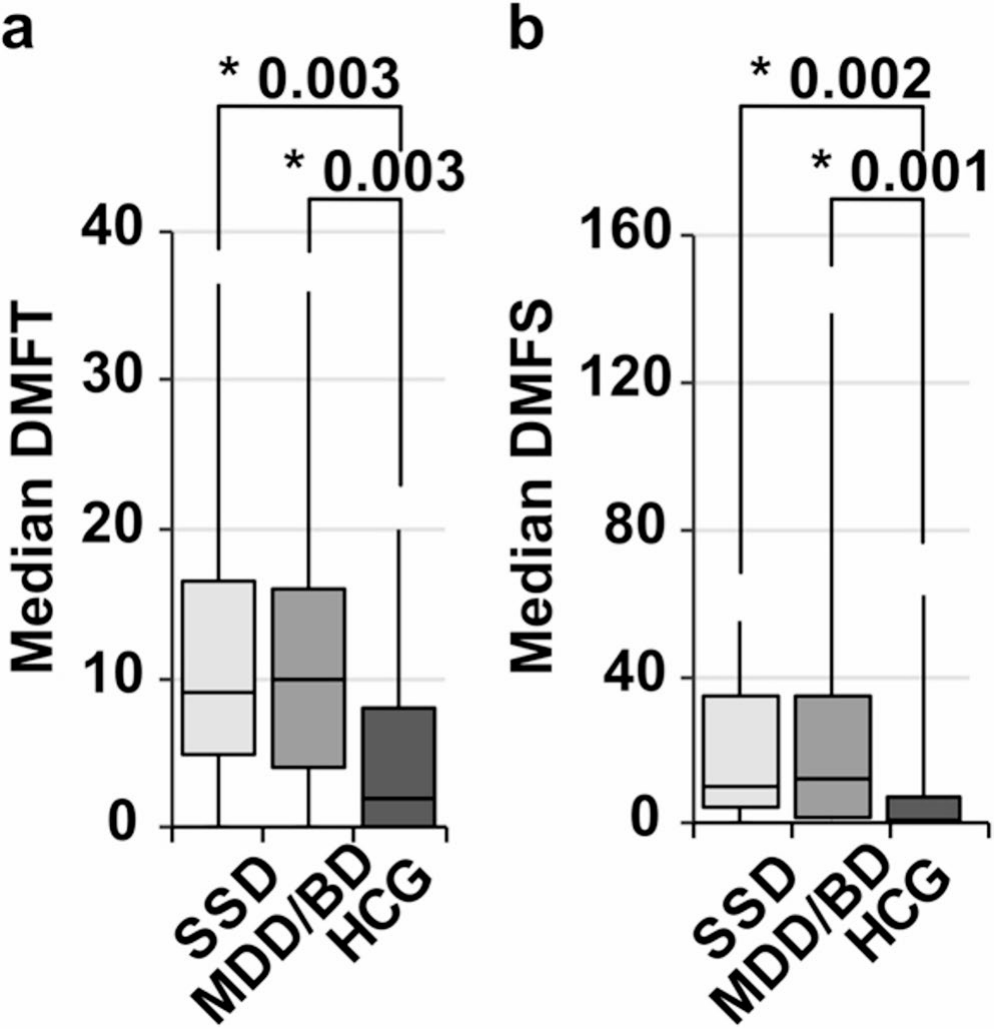
Dental caries comparing SSD and MDD/BD to HCG. SSD = schizophrenia spectrum disorder, CDD/BD = major depression disorder/bipolar disorder, HCG = healthy control group. Median DMFT/DMFS = decayed-missed-filled index for teeth/surfaces. Box-Whiskers-Plots show **a** median DMFT, **b** median DMFS investigated by Kruskal-Wallis test (*p*-values see Table 1) with Dunns post-hoc test (Bonferroni adjustments) shown here

**Fig. 2:**
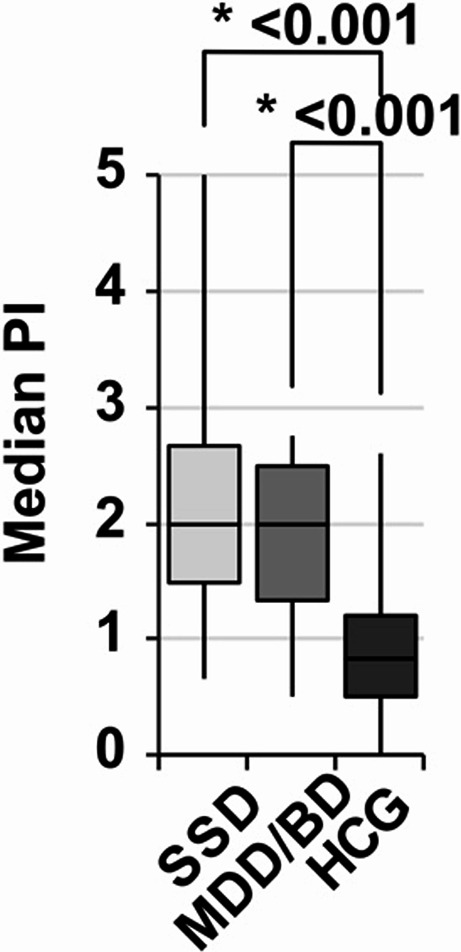
Oral hygiene comparing SSD and MDD/BD to HCG. SSD = schizophrenia spectrum disorder, CDD/BD = major depression disorder/bipolar disorder, HCG = healthy control group. PI = plaque index. Box-Whiskers-Plots show **a** median PI, investigated by Kruskal-Wallis test (*p*-values see Table 1) with Dunns post-hoc test (Bonferroni adjustments) shown here

**Fig. 3:**
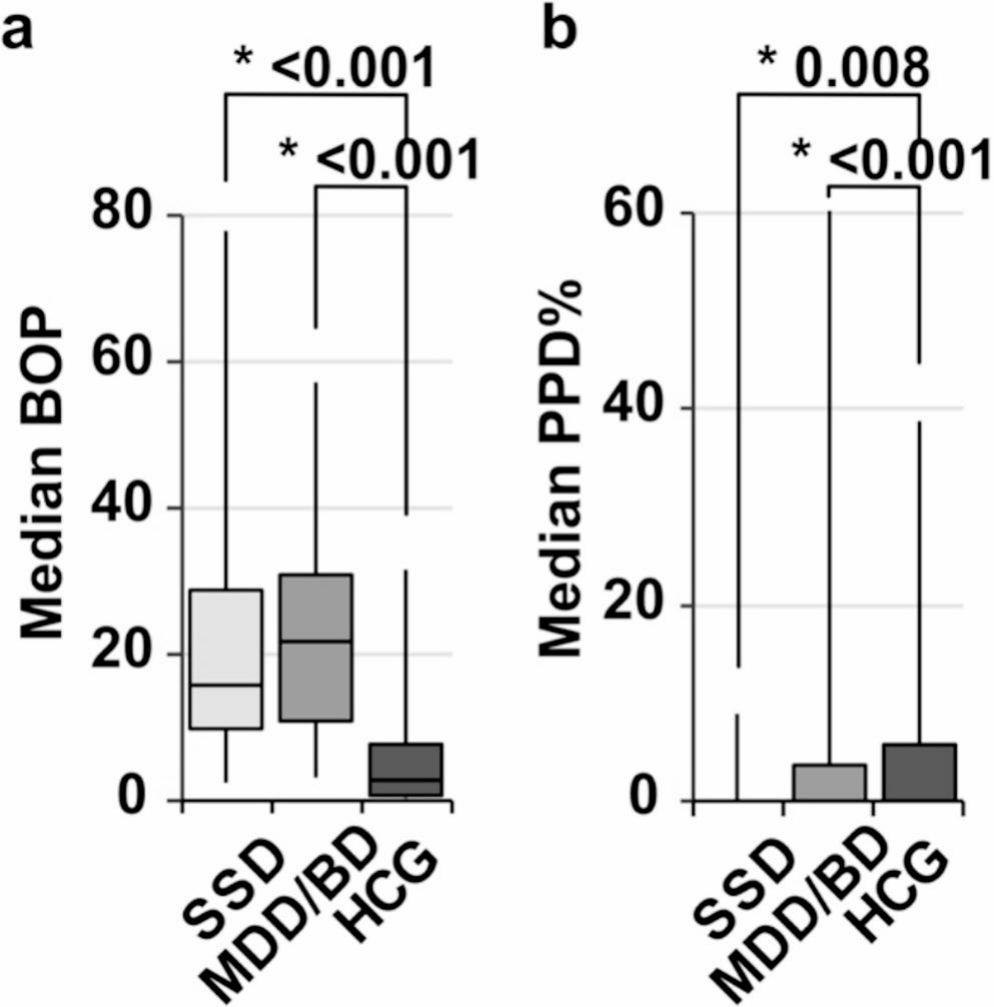
Periodontal health comparing SSD and MDD/BD to HCG. SSD = schizophrenia spectrum disorder, CDD/BD = major depression disorder/bipolar disorder, HCG = healthy control group. BOP = bleeding on probing, PPD% = proportion of periodontal pockets in %. Box-Whiskers-Plots show **a** median BOP and **b** PPD%, investigated by Kruskal-Wallis test (*p*-values see Table 1) with Dunns post-hoc test (Bonferroni adjustments) shown here

Kruskal-Wallis test for the non parametric (Kolmogorow-Smirnow-test) variables age and age at diagnosis, DMFT with DT, MT and FT, DMFS, PI, BOP and PPD% presented as medians (25;75 percentile). BOP N = 110 (reason: N = 6 missing data). Dunn *post-hoc* test with Bonferroni adjustment shown in Figs. 1, 2 and 3

### Association between psychiatric disorders and oral health

In general, patients with SSD or MDD/BD showed a significant higher caries and periodontitis prevalence (Table 1). Significant differences between mentally healthy subjects and the SSD and MDD/BD subgroup were shown in all variables in the *post-hoc* tests (Figs. 1, 2 and 3). The caries parameter median DMFT and median DMFS (Fig. 1) were smaller among mental healthy patients as compared to SSD and MDD/BD as well as the additional oral hygiene parameter PI (Fig. 2). Furthermore, the periodontal parameters SBI, BOP and PPD% revealed significant differences between the healthy controls and the SSD group and the MDD/BD group (Fig. 3). There was no significant difference between SSD and MDD/BD in any of the oral health variables (Table 2**).** Moreover, patients using more than one substance for the treatment of the psychiatric disorder showed significantly poorer medians DMFT, PI (*p* = 0.003), SBI, and BOP scores, while median DMFS and PPD% did not show significant differences compared to patients using only one substance (Table 3).

**Table 2:** Multiple linear regression analysis for associations in oral health

	**DMFT**		**DMFS**		**PI**	
	**95% CI**	***p*** **-value**	**95% CI**	***p*** **-value**	**95% CI**	***p*** **-value**
Sex	3.07 (−5.61-0.54)	**0.018**	−8.49 (−16.38;−0.60)	**0.035**	0.10 (−0.17;0.36)	0.460
Currently Smoking	4.37 (1.38;7.37)	**0.005**	10.26 (0.96;19.57)	**0.031**	0.27 (0.04–0.59)	0.085
Study group	4.62 (1.91;7.39)	**0.001**	12.41(3.96;20.85)	**0.004**	0.96 (0.68;1.24)	**< 0.001**
	**BOP**		**PPD%**			
	**95% CI**	***p*** **-value**	**95% CI**	***p*** **-value**		
Sex	0.57 (−4.94;6.08)	0.837	1.39 (−2.18;4.96)	0.443		
Currently Smoking	3.87 (−2.74;10.49)	0.248	2.49 (−1.72;6.70)	0.244		
Study group	16.12 (10.30;21.93)	**< 0.001**	2.90 (0.92;6.73)	0.135		

Study group (SSD and MDD/BD combined, SSD = schizophrenia spectrum disorder, CDD/BD = major depression disorder/bipolar disorder). DMFT/DMFS = decayed-missed-filled index for teeth/surfaces, PI = plaque index, SBI = sulcus bleeding index, BOP = bleeding on probing, PPD% = proportion of periodontal pockets in %, CI = confidence intervals

Multiple linear regression: Independent variables: sex (index: female, reference: male), currently smoking (index: smoking, reference: non-smoking) and study group (index: SSD + MDD/BD, reference: HCG). Dependent variables: DMFT/DMFS, PI, BOP, PPD% presented as 95% confidence intervals (CI) with lower and upper value and *p*-value

**Table 3:** Medication for mental disorders use and oral health

	Variable	Study group	Monomed	Multimed	*p*-value
	Individuals [N]	64	16	36	
Oral health parameters	DMFT	9.00 (4.25;17.00)	4.00 (0.00;13.00)	11.00 (5.25;20.75)	**0.024**
	DMFS	11.00 5.75)	4.50 (0.00;34.25)	12.50 (5.00;40.50)	0.081
	PI	2.00 (2.00;2.50)	1.33 (1.33;2.08)	2.17 (1.67;2.50)	**0.003**
	BOP	17.90 (9.83;31.05)	10.70 (6.38;14,78)	23.70 (12.20;41.75)	**0.008**
	PPD%	0.00 (0.00;2.68)	0.42 (0.00;1.89)	0.00 (0.00;3.76)	1.000

Study group (SSD and MDD/BD combined, SSD = schizophrenia spectrum disorder, CDD/BD = major depression disorder/bipolar disorder). Monomed = single medication, Multimed = polypharmacy, more than 1 medication from the pharmaceutical groups: typical/atypical antipsychotics, antiepileptics, antidepressants, mood stabilizers, other medications

DMFT/DMFS = decayed-missed-filled index for teeth/surfaces, PI = plaque index, BOP = bleeding on probing, PPD% = proportion of periodontal pockets in %

Mann-Whitney-U-Test for the variables DMFT/DMFS, PI, BOP, PPD% presented as medians (25;75 percentile). N = 12 incomplete information about medications. BOP: SSD/MDD/BD N = 58 (reason N = 6 missing data), Monomed N = 14 (reason: N = 2 missing data), Multimed N = 33 (reason: N = 3 missing data)

### Multivariate analysis

Multiple linear regression analysis revealed a significant association between median DMFT with sex, smoking and having SSD or MDD/BD. PI was associated with SSD or MDD/BD (Table 2). Regarding periodontal health, significant associations were shown between SBI and smoking, and SBI and SSD or MDD/BD as well as BOP and SSD or MDD/BD. No significant associations were observed between the presence of SSD or MDD/BD and PPD% (Table 2).

## Discussion

### Key results

Patients with mental disorders with either SSD or MDD/BD had a significantly higher caries prevalence, as reflected by the DMFT and DMFS index. In line, these patients had also poorer oral hygiene as analyzed with PI, as well as a higher prevalence of periodontitis (Table 2). Herein, significantly more smokers were found among patients in the SSD and MDD/BD groups, potentially further deteriorating oral health parameters (Table 1).

In SSD and MDD/BD the routine use of multiple psychopharmacological medication was associated with poorer oral health, showing a considerably higher prevalence of caries and periodontitis. Since this study did not specifically address this issue, it remains unclear if the individual medication, specifically the use of more than one substance, has a negative impact on oral health. In this regard one might argue that patients with a more complex medication have more severe psychiatric disorders resulting in stronger impairment of self-care attitudes, including regular oral health care.

### Limitations

The small sample size comprising 31 patients with SSD and 33 with MDD/BD impacts on the robustness and generalizability of our findings. The small patient number was mostly caused due to the study design as single-center study with the additional limitations of a study population recruited from a single geographic region. Moreover, 37.5% of patients in SSD and MDD/BD groups refused to participate in a dental examination without any further reason. It can be speculated to align with traumatic previous experiences, neglection of oral health or a poor mental health state that prevents adequate compliance with dental care. Due to the small sample size subgroup analysis of the impact of medications on oral health could not be done herein.

Moreover, healthy control individuals had a younger mean age than the patients with mental conditions reducing the informative value of the study. Taking into account the mean age at the onset of the psychiatric condition patients in both of these study groups presented with a disease history of roughly 10 years when being enrolled into the study. The 65-year age cutoff reduced the impact of comorbidities and polypharmacy but excluded older adults, highlighting a key gap for future research.

Since almost all these patients were recruited at a university hospital one might assume that the majority of mentally affected patients were in fact hospitalized reflecting a more severe psychiatric condition which might have caused significant bias in terms of the representativity of the selected study cohort.

### Interpretation

The association between mental and oral health has been already previously reported [7, 8, 14, 15, 26]. The complexity of this relationship arises from the intricate interplay of multi-layered mechanisms that encompass social, psychological, behavioral and biological domains [7, 10, 22, 24, 29], as well as the additional effect of commonly applied medications [21, 27], acting in both directions. This complex interplay makes the identification of the relevant causes for the association between the mental and oral health status and the determination of their particular effect size rather difficult.

Reduced oral hygiene, as shown by the increased PI might lead to a higher overall caries prevalence as reflected by DMFT and DMFS and a higher prevalence and severity of periodontitis (Table 1). A poorer adherence with routine dental care together with a higher prevalence of dental anxiety among patients with mental disorders might have additionally contributed to this observation. In fact, more than one third of study subjects with a psychiatric diagnosis, despite generally agreeing to participate in this study, declined to join the dental part of the study. Moreover, the MDAS indicated that there were 4 patients with strong dental anxiety in the study group, while there were none among mental healthy subjects further corroborating this assumption.

The current results are in line with data found in a previous case control study on schizophrenia patients with a slightly higher mean age of 40 years [18] presenting with a DMFT of 17.7 compared to 14.2 in healthy controls. A study with hospitalized schizophrenia patients with a mean age of 43.6 years, showed similar results with a DMFT of 18.6 compared to 12.5 in healthy controls [17]. In another cohort study, the DMFT and the DMFS were significantly higher as compared to age and sex matched healthy controls [13]. Additionally, studies considering either male or female patients [29] with severe mental disorders showed a significantly higher caries prevalence [16, 19, 20, 34]. Several previous studies have observed cohorts with a higher mean age taken from populations, with a considerably higher overall caries prevalence than in Germany, where in the age group of 35–44 y the nationwide DMFT is 8.3 (DT = 0.5; MT = 1.0; FT = 6.8 [35]). Therefore, studies that include healthy control groups should be given special consideration when interpreting the current data.

These studies indicate a higher caries prevalence based on an approximately 25% higher DMFT [13, 17, 18] and 31% higher DMFS [13]. Consistent with these findings, our study observed 4–5 times higher DMFT and DMFS scores in participants with SSD and MDD/BD compared to the healthy control group (Table 1). Notably, in our study the stronger prevalence was already evident during the early 30 s of patient life, the typical onset period for psychotic conditions (Table 1**)**. Furthermore, the PI, which reflects the quality of oral hygiene, was significantly higher among individuals with mental disorders. Poor routine oral hygiene measures may contribute to the increased prevalence of common oral diseases, such as caries and periodontitis. This observation aligns, at least partially, with findings from several previous studies [22].

Also the results of stronger prevalence of periodontitis as shown by the increased PPD% in the SSD and MDD/BD group (Table 1) is partially consistent with previous reports on the association between mental and oral health [8, 36]. An almost doubled prevalence of periodontitis was shown among SSD patients compared to a healthy control group [14]. Studies considering exclusively SSD found a stronger clinical attachment level loss in a cohort study compared to healthy patients [22]. A study from 2014 revealed a higher proportion of periodontal pockets as well as poorer gingival and plaque indices correlating with the duration of mental illness in patients aged between 25 and 50 years [26].

Relevant changes of the molecular composition of saliva in patients with mental diseases might probably further amplify the activity of caries and periodontitis [24]. The general association of periodontitis with antipsychotic medication was shown in SSD patients taking xerostomia (dry mouth) inducing medications [28]. Intriguingly, there were no significant differences observed in terms of PPD%, CAL or DMFT between patients and controls.

Regarding medications, the current results may reflect the cumulative effects of the combined use of several different therapeutic substances on oral health. This impact could be attributed to various factors, including xerostomia, which is a common side effect of many psychiatric medications such as antipsychotics, antidepressants, and mood stabilizers [21, 27, 28]. Xerostomia can reduce the protective effects of saliva, increasing the risk of dental caries and periodontal disease [21, 27, 28]. Additionally, a more complex drug therapy may alter the oral microbiota, disrupting the balance of the oral microbiome ultimately affecting oral health issues [37, 38]. Furthermore, challenges in maintaining oral hygiene, which may be exacerbated by the sedative or cognitive effects of some medications, can further enhance these risks [39]. The current results clearly underpin the importance of individual oral health care for patients with mental diseases, particularly for patients under complex medication comprising different substances. While one study has shown limited effects of individualized tooth brushing training in children and adolescents (aged 6–17) with psychiatric conditions, oral health-related quality of life was strongly influenced by the personal attention, support, and engagement provided [40]. This highlights the critical role of motivational and relational factors in oral health outcomes. Furthermore, patients with psychiatric conditions of a Norwegian study reported often significant barriers to seeking dental care, including shame, perceived inferiority, and fear of not being taken seriously. These feelings are frequently accompanied by mistrust and a fear of burdening or disappointing dental professionals, leading patients to suppress their needs even in distress [41]. Building on the finding that a significant proportion of dentists in Germany even with expertise in pediatric dentistry report lacking knowledge and experiencing stress when treating patients with special needs, there might be need to strengthen special care dentistry education within dental training programs [42, 43]. Consequently, these findings open important avenues for future research into innovative motivational strategies tailored to high-risk groups—exploring not only their effectiveness and implementation but also their accessibility, including the potential use of digital media and technology-based interventions.

### Generalizability

While the study data are roughly in line with previous studies, the generalizability of observations of this monocentric patient group from a German cohort including a small number of cases only appears limited. While the current study has limited generalizability, the consistency with previous observations - despite differences in design and baseline conditions - corroborates a common trend of increased prevalence and intensity of both, caries periodontitis among patients with mental diseases.

## Conclusion

This study highlights an increased demand for oral health care among mentally diseased patients. Poorer oral hygiene and higher caries and periodontal prevalence where observed in SSD and MDD/BD patients. The influence in increasing the prevalence by multiple medications must be taken into account when interpreting the current results. The usefulness or efficacy of personalized oral hygiene training and routine dental care were not investigated.

## Electronic supplementary material

Below is the link to the electronic supplementary material.

Supplementary Material 1

## Data Availability

.The data that support the findings of this study are available from the corresponding author UW upon reasonable request.
