# Supplementary material for: Association of psychosis and oral health: case-control study
Source: Clin Oral Investig. 2025 Aug 6;29(9):397. doi: 10.1007/s00784-025-06463-6 (PMC12325445; doi:10.1007/s00784-025-06463-6)
Supplement: Supplementary file 1 — Supplementary Material 1 [file 784_2025_6463_MOESM1_ESM.docx]

**Supplemental Files**


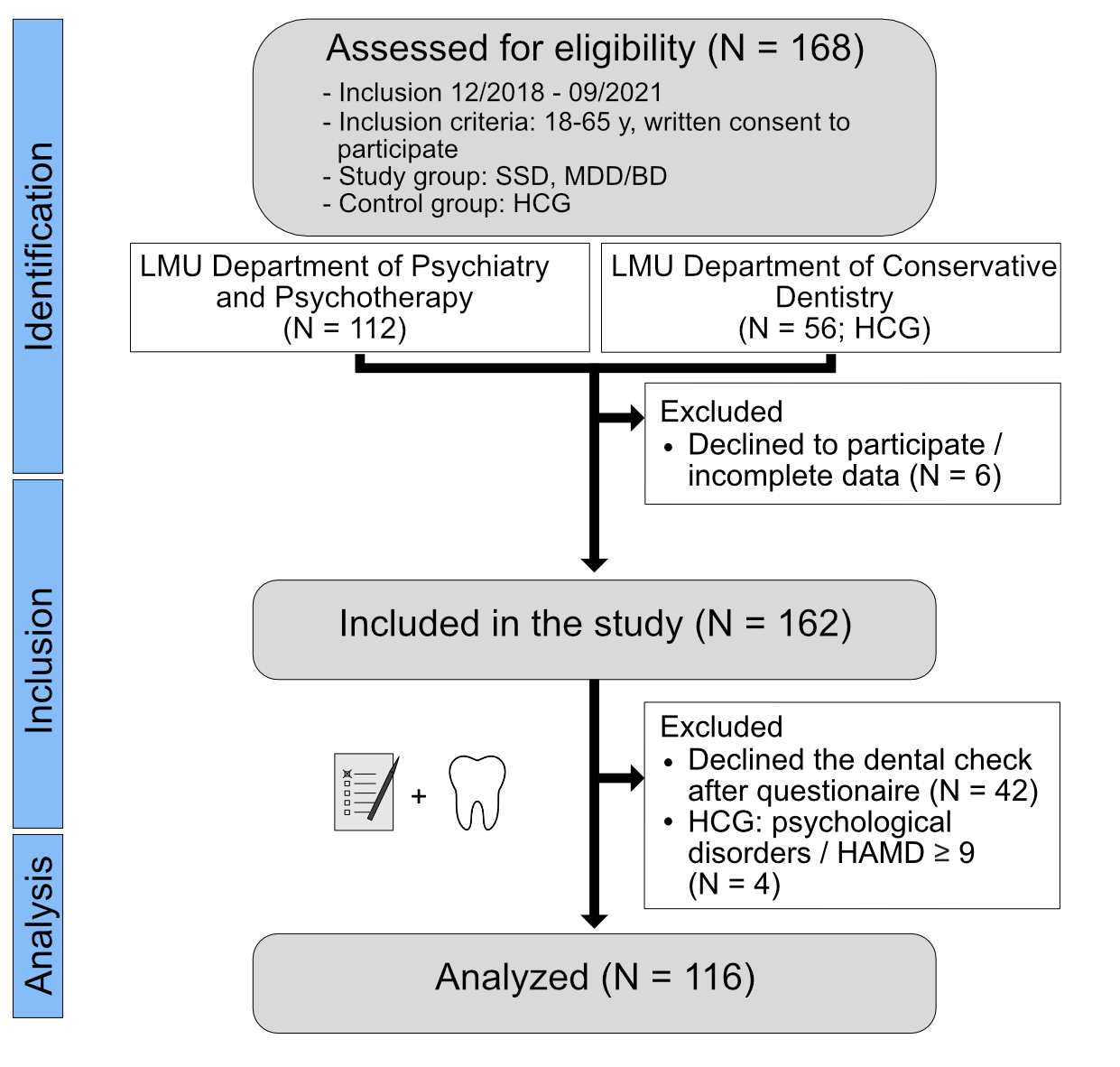


**Supplements 1 - STROBE guideline flow chart of participants - study design.**N = number, SSD = schizophrenia/schizophrenia spectrum disorder, MDD/BD = major depressive disorder/bipolar disorder, HCG = healthy control group, HAMD = Hamilton Rating Scale for Depression (screening: 0-7 = not depressed, 8 or more = increasing levels of depression).

**Supplements 2 – Oral health parameters comparing SSD + MDD/B.**

|  | **Variable** | **All** | **SSD+ MDD/BD** | **HCG** | **p-value** |
| --- | --- | --- | --- | --- | --- |
| **Oral health parameters** | Median DMFT^1^ | 7 (1;13) | 9 (4;17) | 2 (0;8) | **<0.001** |
|  | DT^1^ | 0 (0;3) | 1 (0;4) | 0 (0;0) | **<0.001** |
|  | MT^1^ | 0 (0;1) | 0 (0;2) | 0 (0;0) | **<0.001** |
|  | FT^1^ | 4 (1;9) | 6 (1;10) | 2 (0;7) | **<0.001** |
|  | Median DMFS^1^ | 5 (0;23) | 11 (2;36) | 1 (0;7) | **<0.001** |
|  | PI [grade]^1^ | 1 (1;2) | 2 (2;3) | 1 (1;1) | **<0.001** |
|  | BOP [%]^1^ | 9 (3;24) | 18 (10;31) | 3 (0;8) | **<0.001** |
|  | PPD% [%]^1^ | 0 (0;1) | 0 (0;3) | 0 (0;0) | **<0.001** |
| SSD = schizophrenia spectrum disorder, CDD/BD = major depression disorder/bipolar disorder, HCG = healthy control group. DMFT/DMFS = decayed-missed-filled index for teeth/surfaces, PI = plaque index, BOP = bleeding on probing, PPD% = proportion of periodontal pockets in %.  ^1^ Data shown as medians with (25;75 percentile) following non normality distribution tested by Kolmogorow-Smirnow-test. | | | | | |

Kruskal-Wallis test for the non parametric (Kolmogorow-Smirnow-test) DMFT with DT, MT and FT, DMFS, PI, BOP and PPD% presented as medians (25;75 percentile). BOP N = 110 (reason: N = 6 missing data).
